# Wip1 regulates the immunomodulatory effects of murine mesenchymal stem cells in type 1 diabetes mellitus via targeting IFN-α/BST2

**DOI:** 10.1038/s41420-021-00728-1

**Published:** 2021-10-29

**Authors:** Na Zhou, Weijiang Liu, Wei Zhang, Yuanlin Liu, Xue Li, Yang Wang, Rongxiu Zheng, Yi Zhang

**Affiliations:** 1 Department of Experimental Hematology and Biochemistry, Beijing Institute of Radiation Medicine, Beijing, 100850 China; 2 Department of Pediatrics, Tianjin Medical University General Hospital, Tianjin, 300052 China; 3 Department of Medical Administration, The Sixth Medical Center of PLA General Hospital, Beijing, 100048 China

**Keywords:** Mesenchymal stem cells, Autoimmune diseases

## Abstract

Mesenchymal stem cells (MSCs) show significant therapeutic effects in type 1 diabetes mellitus (T1DM) as regulating the inflammatory processes. However, little is known about the detailed process of MSCs immunosuppression in T1DM. In this study, we investigated the effects of wild-type p53-induce phosphatase 1 (Wip1) on regulating MSCs immunosuppressive capacities in T1DM mice. We found that Wip1 knockout (Wip1^−/−^) MSCs had lower therapeutic effects in T1DM mice, and displayed weaker immunosuppressive capability. In vivo distribution analysis results indicated that

Wip1^−/−^MSCs could home to the damaged pancreas and increase the expression of tumor necrosis factor-α (TNF-α), interleukin-17a (IL-17a), interferon-α(IFN-α), IFN-β, and IFN-γ, while decrease the expression of IL-4 and IL-10. Moreover, we confirmed

Wip1^−/−^MSCs exhibited weaker immunosuppressive capacity, as evidenced by enhanced expression of bone marrow stromal cell antigen 2(BST2) and IFN-α. In conclusion, these results revealed Wip1 affects MSCs immunomodulation by regulating the expression of IFN-α/BST2. Our study uncovered that Wip1 is required to regulate the therapeutic effects of MSCs on T1DM treatment, indicating a novel role of Wip1 in MSCs immunoregulation properties.

## Introduction

Type 1 diabetes mellitus (T1DM), an autoimmune disease induced by multiple factors, causes pancreatic infiltration of T lymphocyte and destruction of β islet cells, which ultimately leads to a significant decline in insulin release [1–3]. Currently, mesenchymal stem cells (MSCs) have been extensively utilized in treating T1DM and its complications as they contribute to alleviate inflammation and reduce the apoptosis of β islet cells [4, 5]. However, the molecular cues of MSCs in T1DM are still unclear.

Wild-type p53-induce phosphatase 1 (Wip1), encoded by the *PPM1D* gene, is a serine/threonine phosphatase newly identified in wild-type p53-induced phosphatase of the PP2C family [6]. It has been reported to be closely associated with tumorigenesis, cell proliferation, as well as development and aging processes [7]. Meanwhile, it played important role in regulating the function of immune cells [8–10**]**. Recently, several studies demonstrated that Wip1 could modulate MSCs migration, proliferation, and senescent growth arrest [11, 12]. Wip1 depletion in mouse embryonic fibroblasts could lead to the reduction of insulin in diabetes [10, 13]. Also, Wip1 could regulate islet cell proliferation and regeneration through modulating the p38 MAPK pathway [14]. These indicated Wip1 was essential for the biological characteristics of MSCs and islet cells. Nevertheless, the effects of Wip1 on the immunomodulatory function of MSCs need to elucidation.

To investigate the effects of Wip1 on immunosuppressive properties of MSCs and the therapeutic effects of T1DM, we isolated MSCs from Wip1^−/−^mice and injected into T1DM mice. Wip1^−^/^−^MSCs showed weak anti-inflammatory activity and impaired therapeutic efforts in T1DM mice. We also found that BST2 contributed to the high expression of interferon-α (IFN-α) in Wip1^−/−^MSCs, which was an important factor in the pathogenesis of T1DM. Importantly, administration of Wip1^−/−^MSCs to T1DM mice potently promoted IFN-α expression and aggravation of inflammatory responses in the pancreatic microenvironment. In a word, we uncovered that Wip1 played vital roles in the therapeutic effects of MSCs in T1DM mice, which provided a novel mechanism for understanding the immunosuppressive capacity of MSCs.

## Results

### Isolation and identification of Wip1^−/−^MSCs from murine compact bones

**Fig. 1:**
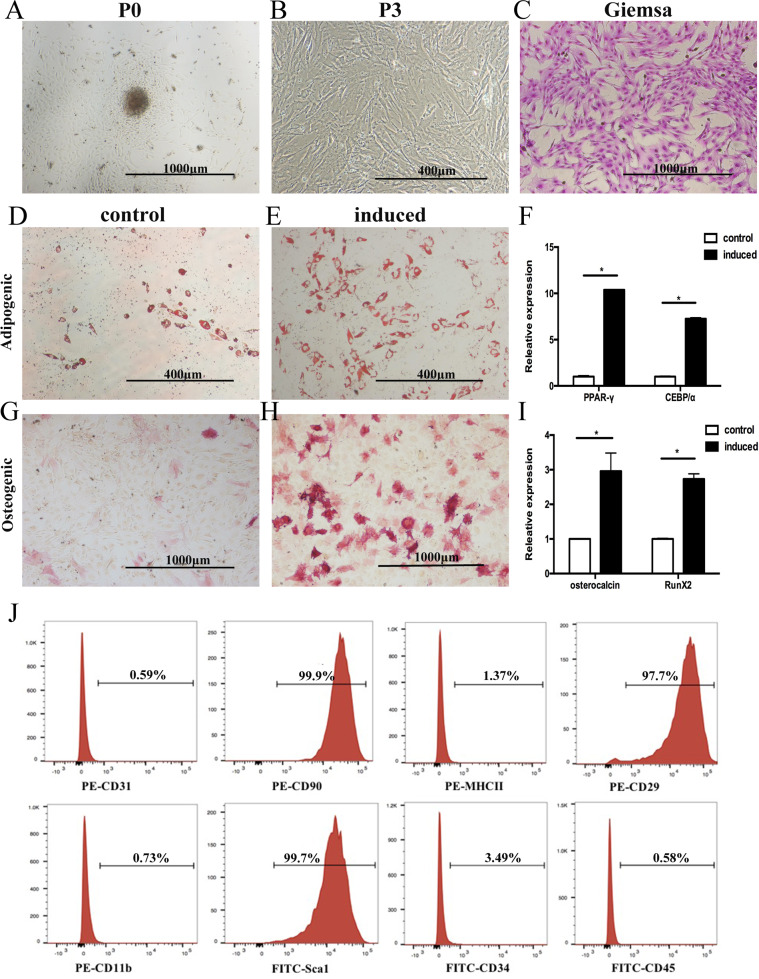
Isolation and identification of Wip1^−/−^ MSCs from murine compact bones. **A** Nucleated cells isolated from compact bones of Wip1^−/−^ mice became adherent 72 h after seeding. **B** The adherent cells (P3) displayed fibroblast-like morphology. The adherent cells were stained with Wright-Giemsa. **C** Multilineage differentiation potential of compact bone-derived adherent cells in Wip1^−/−^mice was assessed by inducing adipogenic or osteogenic capacities in vitro. **D**, **E** Adipogenesis differentiation was indicated by the presence of lipid drops stained with oil red O. **G**, **H** Osteogenic differentiation was shown by the intracytoplasmic accumulation of alkaline phosphatase. **F**, **I** The expression of *PPAR-γ* and *CEBPɑ* in the adipogenic induction group and *Osteocalcin* and *Runx*2 in the osteogenic induction group was detected by qPCR (*n* = 3). **J** Immunophenotyping of culture-expanded adherent cells from Wip1^−/−^ mice compact bones-derived adherent cells were analyzed by flow cytometry. *P < 0.05, compared with control group.

MSCs were isolated from Wip1^+/+^ and Wip1^−/−^ mice by cultivating the digested compact bone. Fibroblast-like cells sprouted from the bone fragments and adhered to the flask 48 h (Fig. 1A). These adherent cells could be readily expanded in vitro by successive cycles of trypsinization, seeding, and culture every 3 days without visible morphologic alteration (Fig. 1B, C).

We then determined the adipogenic and osteogenic differentiation of Wip1^−/−^cells in vitro. As shown in Fig. 1D, E, many Oil Red-O-positive lipid droplets were available in murine Wip1^−/−^MSCs. Consistent with histochemical staining results, the mRNA expression of *C/EBPα* and *PPAR-γ* in Wip1^−/−^MSCs showed elevation (Fig.1F). Under osteogenic conditions, the cells from Wip1^−/−^mice displayed alkaline phosphatase activity (Fig. 1G, H). In line with the results of differentiation assays, qPCR demonstrated transcriptional expression of *Osteocalcin* and *Runx2* under specific osteogenic cultures in adherent cells (Fig. 1I). The immunophenotype of the Wip1^−/−^ adherent cells was assessed by flow cytometry. The culture-expanded adherent cells were positive for CD90, CD29, and Sca-1 but were negative for CD31, MHCII, CD11b, CD34, and CD45 (Fig. 1J). The morphologic, immunophenotypic, and differentiation assays strongly indicated that the adherent cells isolated from Wip1^−/−^mice were MSCs. In addition, Wip1^+/+^MSCs showed similar biological characteristics (data not shown).

### Therapeutic effect of Wip1^−/−^MSCs in T1DM mice

**Fig. 2:**
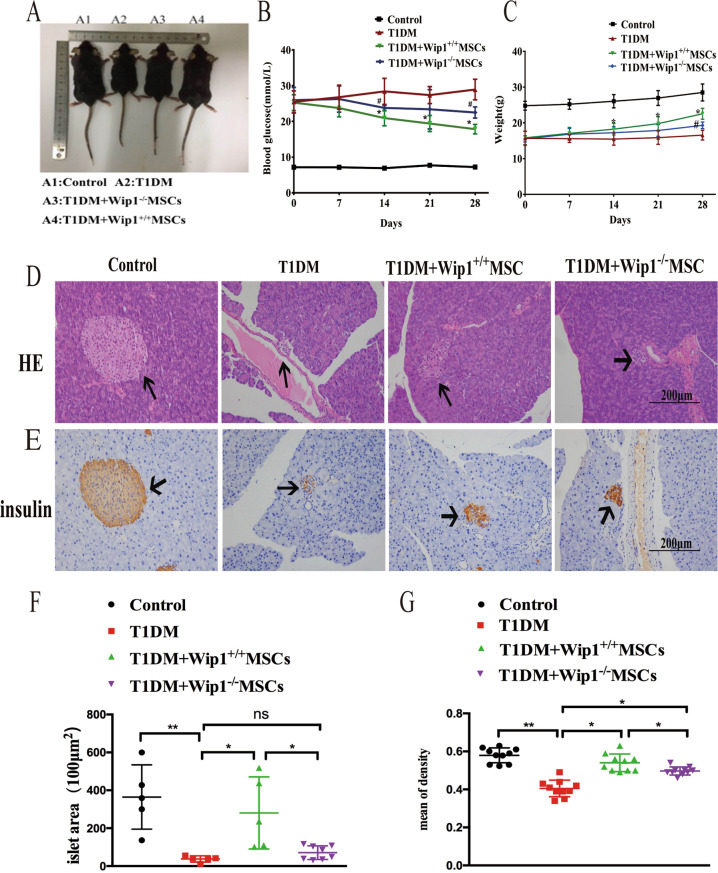
Wip1 knockout impaired the therapeutic efficacy of MSCs in T1DM mice. **A** The body size of mice after infusion of Wip1^+/+^MSCs and Wip1^−/−^ MSCs. **B**, **C** The blood glucose and the weight curves. **D**, **E** Pathological damages in pancreatic tissue by H&E staining and immunohistochemical staining. **F**, **G** The islet area and the mean density of insulin-positive cells in pancreatic tissues treated with Wip1^−/−^ MSCs were smaller than that of the Wip1^+/+^MSCs group. (*n* = 8, three independent experiments), ^**#**^*P* < 0.05, *P < 0.05, **P < 0.01.

To assess the therapeutic effect of Wip1^−/−^ MSCs in T1DM mice, Wip1^+/+^MSCs and Wip1^−/−^MSCs were intravenously administered to STZ-induced T1DM mice. As shown in Fig. 2A, the body size in Wip1^−/−^MSCs mice was smaller compared with Wip1^+/+^MSCs mice. The blood glucose levels were higher in Wip1^−/−^MSCs compared with the Wip1^+/+^MSCs group (Fig. 2B). Additionally, the slow gain in weight caused by hyperglycemia was controlled by MSCs infusion. The average body weight of the Wip1^+/+^MSCs group was larger than the Wip1^−/−^ MSCs group (Fig. 2C).

To further evaluate whether Wip1^−/−^MSCs infusion could reduce the pathological damages in T1DM mice, the histological examination of islet tissues was performed by H&E staining and immunohistochemical staining on day 28. As shown in Fig. 2D, E, islets of the Wip1^−/−^MSCs group exhibited mild inflammation. Additionally, there were a few islet areas with preserved islet morphology, indicating that Wip1^−/−^MSCs infusion could prevent the destruction of islets in T1DM. Moreover, the islet area and the mean density of insulin-positive cells in pancreas treated with Wip1^+/+^MSCs were larger than that of the Wip1^−/−^MSCs group, despite that the effects of Wip1^−/−^MSCs were better than T1DM mice (Fig. 2F, G). These findings indicated the therapeutic effects of Wip1^−/−^MSCs were weaker than Wip1^+/+^MSCs in T1DM mice.

### Wip1^−/−^MSCs failed to reduce the inflammatory response in T1DM mice

**Fig. 3:**
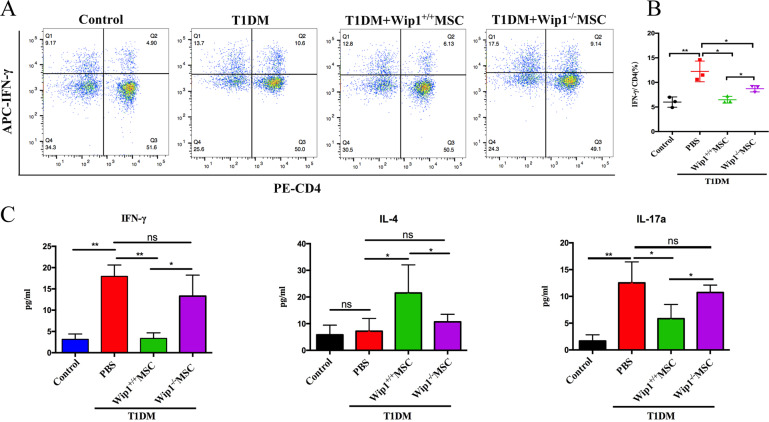
Wip1^−/−^ MSCs exhibited lower immunosuppression response in T1DM mice. **A** Frequency of CD4^+^IFN-γ^+^ Th1 cells was analyzed by flow cytometry in MSCs-treated T1DM mice. **B** The quantitative results for CD4^+^IFN-γ^+^ Th1 cells in splenic lymphocytes. **C** Serum IFN-γ, IL-17a, and IL-4 were detected by ELISA. (*n* = 7, three independent experiments) ns, not significant, *P < 0.05, **P < 0.01.

Previous studies indicated that the immunoregulation of MSCs was closely involved in the Th1 inflammatory processes in T1DM [15, 16]. To investigate whether Wip1 regulated the immunomodulatory function of MSCs in T1DM. Flow cytometry was used to detect IFN-γ in Th1 cells (CD4^+^IFN-γ^+^ T cells) of spleen lymphocytes in each group. As shown in Fig. 3A, B, the percentage of Th1 cells (CD4^+^IFN-γ^+^ T cells) in the T1DM group was significantly higher than that in other groups (*P* < 0.01). Both Wip1^−/−^MSCs and Wip1^+/+^MSCs infusion significantly decreased the percentage of Th1 cells (*P* < 0.05). However, the percentage of Th1 cells in the Wip1^−/−^MSCs group was lower than that of the T1DM group and was higher than that of the Wip1^+/+^MSCs group (*P* < 0.05).

Subsequently, we investigated whether Wip1 could affect pro-inflammatory factors in T1DM mediated by MSCs infusion. Compared with the Wip1^+/+^MSCs group, serum IFN-γ and IL-17a increased and IL-4 decreased in the Wip1^−^^/−^MSCs infusion group (Fig. 3C). These data indicated that Wip1 is critical for the immunomodulatory activity of MSCs in T1DM mice.

### Wip1 interacted with BST2

**Fig. 4:**
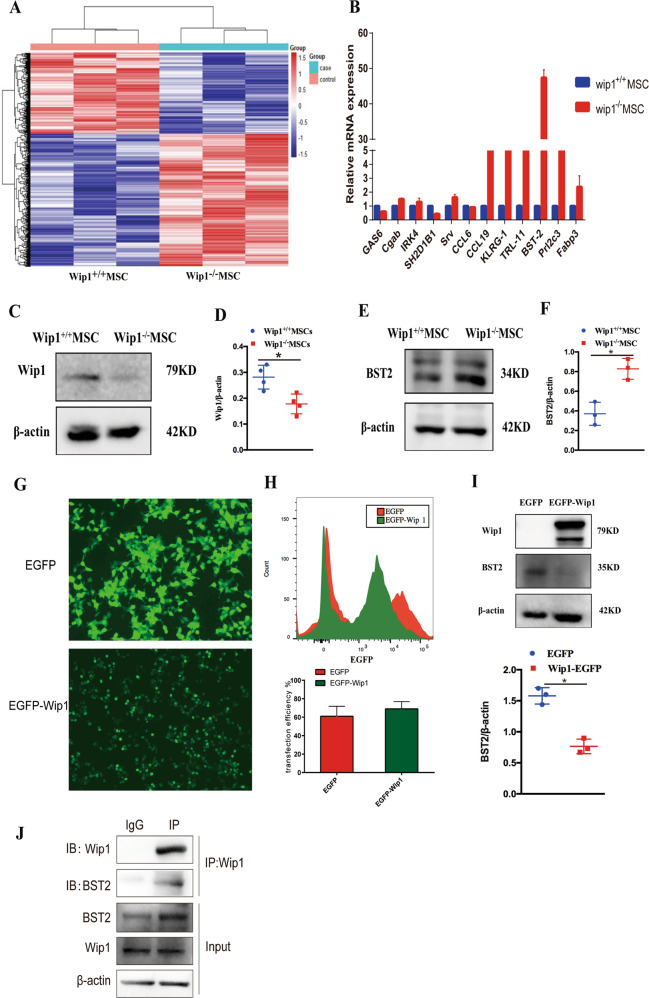
Wip1 interacted with BST2. **A** The RNA transcripts heatmap of Wip1^+/+^MSCs and Wip1^−/−^ MSCs was displayed by Microarray analysis. **B** The upregulated genes in Wip1^+/+^MSCs and Wip1^−/−^MSCs were determined by qPCR. **C**–**F** Western blot was used to confirm the protein expression of Wip1 and BST2 in Wip1^+/+^MSCs and Wip1^−/−^ MSCs. **G** Wip1-EGFP and EGFP plasmids were transfected into 293 T cells, and the positive cells were observed by fluorescence microscope. **H** The transfection efficiency of Wip1-EGFP and EGFP plasmids in 293 T cells was detected by flow cytometry. **I** Western blot assay was utilized to determine the endogenous interaction between Wip1 and BST2 in 293 T cells. **J** Co-IP assay was used to detect the interaction between endogenous Wip1 and BST2 in MSCs cells. *P < 0.05.

To investigate the involvement of Wip1 in MSCs immunomodulatory activity, we then determined the gene expression profiles in Wip1^+/+^MSCs and Wip1^−/−^MSCs by microarray analysis. Bioinformatics methods were used to analyze the microarray results, which predicted that 23 genes were associated with immunomodulatory responses. Among the upregulated genes in Wip1^−/−^MSCs, *BST2* was identified to be related to inflammatory processes, and highly expressed in Wip1^−/−^MSCs (Fig. 4A, B and Table S1). To further explore whether *BST2* was upregulated in Wip1^−/−^MSCs, we examined the expression of *BST2* in Wip1^+/+^MSCs and Wip1^−/−^MSCs. As shown in Fig.4C–F, compare with Wip1^+/+^MSCs, the protein level of BST2 increased in Wip1^−/−^ MSCs.

Subsequently, we further investigated whether Wip1 interacted with BST2. The 293 T cells were transfected with Wip1-EGFP, followed by determining the expression of Wip1 and BST2. EGFP expression indicated high transduction efficiency, which showed a transfection efficiency of 61 ± 10.81% and 69 ± 7.93% for the EGFP group and Wip1-EGFP group, respectively (Fig. 4G, H). As shown in Fig. 4I, after overexpression of Wip1 in 293 T cells, thereby the BST2 expression decreased. Furthermore, endogenous CO-IP demonstrated that Wip1 and BST2 interact in MSCs (Fig. 4J). Collectively, these results suggest that Wip1 is associated with BST2 in MSCs.

### BST2 promote IFN–α expression in Wip1^−/−^MSCs

**Fig. 5:**
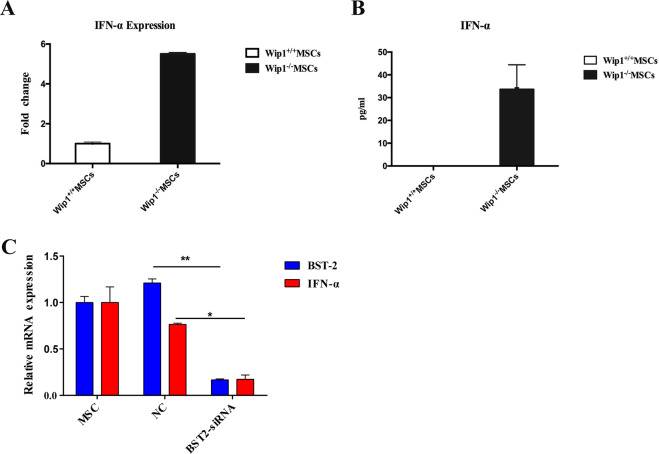
BST2 promotes *IFN–α* expression in Wip1^−/−^MSCs. **A**, **B** The mRNA and protein levels of IFN-α in Wip1^+/+^MSCs and Wip1^−/−^MSCs were detected by qPCR and ELISA. **C** Wip1^−/−^ MSCs were transfected with BST2 siRNA, and the mRNA level of BST2 and IFN-α in Wip1^−/−^ MSCs were measured by qPCR 48 h after transfection. Three independent experiments, *P < 0.05, **P < 0.01.

BST2 is activated plasma cell-derived dendritic cells (pDC) and promoted IFN-α expression [17]. IFN-α is involved in the regulation of the biological characteristics of MSCs and promoted the inflammatory response of T1DM [18–20]. Therefore, we explored whether BST2 could affect the regulation of Wip1^−/−^MSCs on the inflammatory process in T1DM mice via IFN-α. As shown in Fig. 5A, the mRNA expression of *IFN-α* in Wip1^−/−^MSCs was significantly higher than that of Wip1^+/+^MSCs. Compared with Wip1^+/+^MSCs, IFN-α in Wip1^−/−^MSCs showed a dramatic increase (Fig. 5B). To investigate whether IFN-α was dependent on the BST2 expression in Wip1^−/−^MSCs, we transfected Wip1^−/−^MSCs with *BST2*-siRNAs or negative control. The results showed that *BST2* knockdown in Wip1^−/−^MSCs could significantly downregulate the expression of IFN-α (*P* < 0.05, Fig. 5c). This implied that the knockdown of BST2 could effectively inhibit the expression of IFN-α. On this basis, we demonstrated that BST2 was a key gene for regulating IFN-α expression in Wip1^−/−^MSCs.

### IFN-α secreted by Wip1^−/−^MSCs aggravated inflammatory response in the pancreatic microenvironment

**Fig. 6:**
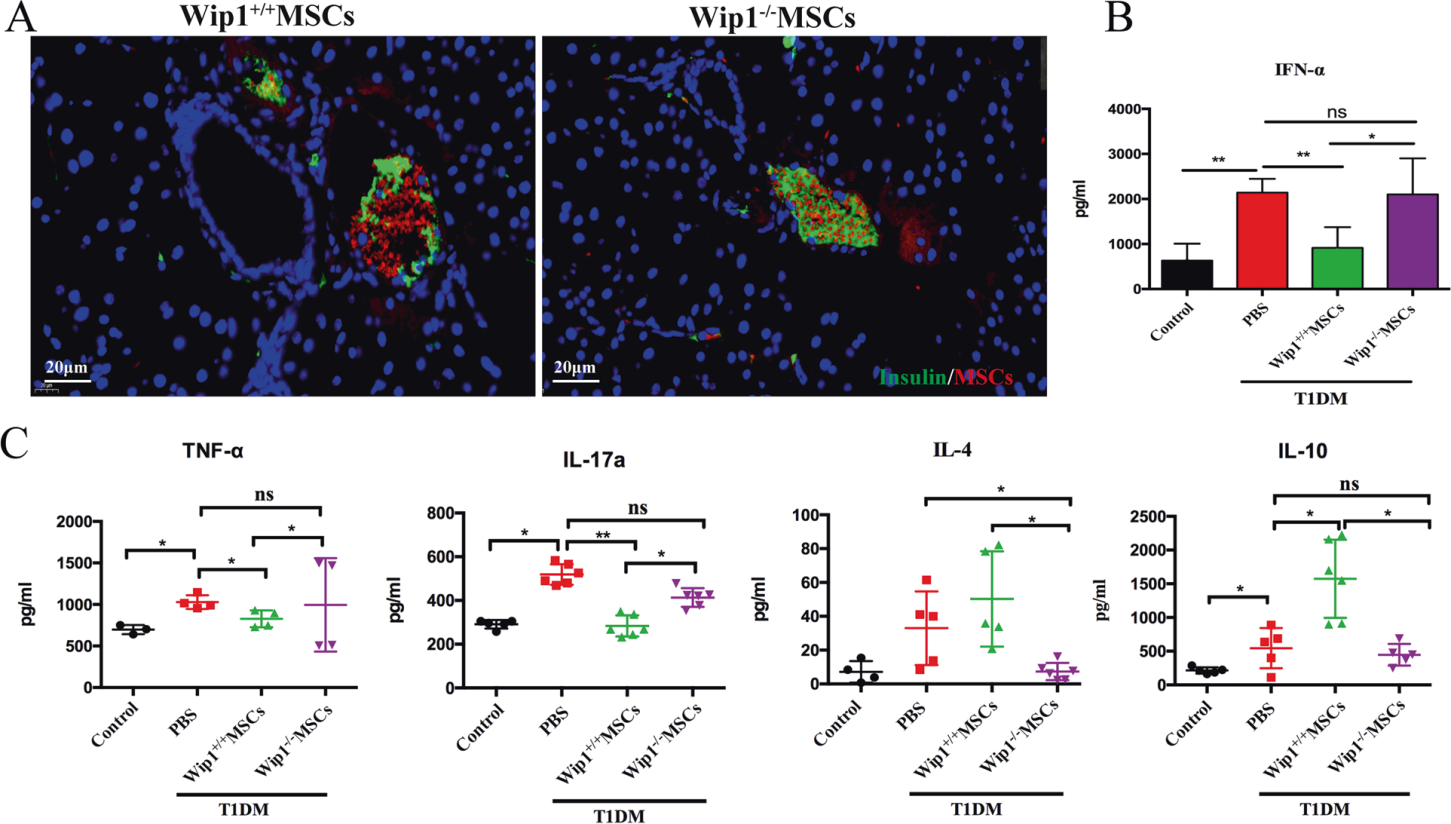
IFN-α secreted by Wip1^−/−^MSCs enhanced the inflammatory response in the pancreatic microenvironments of T1DM mice. **A** Immunofluorescence staining of pancreatic microenvironment including insulin (green), MSCs (red), and nuclei (blue) showed the size change of insulin and homing of MSCs in T1DM mice with an infusion of Wip1^+/+^MSCs and Wip1^−/−^MSCs. **B**, **C** Expression of IFN-α, TNF-α, IL-17a, IL-4, and IL-10 in the pancreas by ELISA. Scale bar, 20 μm. (*n* = 7, three independent experiments) ns, not significant, *P < 0.05, **P < 0.01.

Several studies had shown that MSCs preferentially homed to the sites of tissue damages, where they enhanced wound healing and were involved in modulating the balance of inflammatory response [21]. To determine whether Wip1^−/−^MSCs could migrate to the damaged sides in the pancreas, red fluorescence stained Wip1^−/−^MSCs and Wip1^+/+^MSCs were administrated into T1DM mice. After staining for 72 h, red fluorescence occurred around blood vessels in pancreatic tissues (Fig. 6A), which indicated that Wip1^−/−^ MSCs could home to the pancreatic tissues in T1DM mice.

IFN-α was indicated to play important roles in T1DM immune disorders. To explore whether IFN-α increased due to Wip1^−/−^MSCs infusion, the expression of IFN-α in the pancreatic supernatant of T1DM mice was detected by ELISA. The expression of IFN-α in the pancreatic supernatant of the Wip1^−/−^MSCs group showed a significant increase (2103.14 ± 797.1 pg/mL) compared with the control group and Wip1^+/+^MSCs group (*P* < 0.05). Compared with the T1DM model group, there was no significant difference. These data demonstrated that Wip1^−/−^MSCs could promote IFN-α secretion after homing to pancreatic tissue (Fig. 6B). Besides, we further examined the expression of *IFN-α*, *IFN-β*, and *IFN-γ* in splenic lymphocytes. The expression of *IFN-α, IFN-β*, and *IFN-γ* in the Wip1^−/−^MSCs group was higher than that of the Wip1^+/+^MSCs group, which indicated that Wip1^−/−^MSCs also could promote the expression of *IFN-α, IFN-β*, and *IFN-γ* in spleen lymphocytes, and further aggravate the inflammatory response (Fig. S1).

The expression of inflammatory cytokines (e.g., TNF-α, IL-17a, IL-4, and IL-10) in the pancreas supernatant was measured by ELISA. The expression of TNF-α (1027.87 ± 82.9 pg/ml) and IL-17a (519.02 ± 47.01 pg/ml) in the T1DM group were significantly higher than those in other groups (*P* < 0.05). After treating with Wip1^+/+^MSCs, the expression of TNF-α (827.03 ± 102.08 pg/ml) and IL-17a (283.53 ± 48.26 pg/ml) showed significant decline compared with those in T1DM group (*P* < 0.05), while the expression of TNF-α (995.24 ± 562.61 pg/ml) and IL-17a (413.33 ± 42.98 pg/ml) in Wip1^−/−^MSCs treatment group was significantly higher than those in Wip1^+/+^MSCs group (*P* < 0.05). The expression of IL-4 (50.25 ± 28.14 pg/ml) and IL-10 (1575 ± 579.38 pg/ml) in the Wip1^+/+^MSCs group was significantly higher than those of other groups (*P* < 0.05). IL-4 (7.35 ± 3.5 pg/ml) and IL-10 (447.5 ± 160.17 pg/ml) expression in the Wip1^−/−^MSCs group was significantly lower than those in the Wip1^+/+^MSCs group (*P* < 0.05). These data indicated that IFN-α secreted by Wip1^−/−^MSCs could aggravate the inflammatory response in the T1DM mice pancreatic microenvironment (Fig. 6C).

## Discussion

T1DM is a public health challenge worldwide with a hallmark of autoimmune attack of β-cells leading to specific loss of the insulin-secreting cells [22]. Previous clinical studies demonstrated that moderate immunosuppression in T1DM could prevent further loss of insulin production, which then attenuated the clinical symptoms [23–25]. With the capacity to modulate immune responses, MSCs have been widely employed to treat various inflammatory diseases including T1DM [25–27], however, the potential mechanisms remain largely elusive.

The study indicated wip1 effected the immunomodulatory functions of MSCs in T1DM via targeting IFN-α/BST2. Also, we provided a new mechanistic insight in the Wip1 regulated the immunomodulatory function of MSCs based on in vitro and in vivo evidence. Wip1^−/−^MSCs had impaired therapeutic effects in the T1DM model, including failed to decrease inflammatory response and increase IFN-α in the pancreas microenvironment.

Wip1 is expressed in hematopoietic progenitors, stem cells, neutrophils, macrophages, B and T lymphocytes [28, 29]. Recent studies demonstrated that Wip1 could regulate immune cells’ proliferation, activation, and differentiation activities [8, 28, 30, 31]. Moreover, Wip1 was crucial for the modulation of MSCs migration, but little is known about the effects of Wip1 on the immunosuppression capacity of MSCs. In this study, we showed that the therapeutic effect of Wip1^−/−^MSCs on T1DM in mice was significantly lower than that of the Wip1^+/+^MSCs group. Wip1^−/−^MSCs triggered no decline of the Th1 frequency, and the level of serum IFN-γ and IL-17a was nearly the same in T1DM mice. Previously, Th1 pro-inflammatory autoimmune response led to a failure of immune tolerance to β-cells [25], while MSCs decreased the frequency of Th1 cells and reduced IFN-γ and IL-17a in T1DM. Therefore, the immunosuppressive effects of MSCs on T1DM were more likely to depend on Wip1. However, little is known about the molecular mechanism involved in the MSCs immunomodulation by Wip1. The Wip1^−/−^MSCs microarray assay showed that the BST2 expression increased. Moreover, Western blot and CO-IP assay indicated that Wip1 not only inhibits the expression of BST2 but also Wip1 and BST2 interact in MSCs. Previous studies reported that the expression of BST2, serving as a surface protein involved in viral vesicle budding in response to interferons [32, 33]. Our data also indicated that *IFN-α* in Wip1^−/−^MSCs was significantly upregulated in protein and mRNA levels. Moreover, the BST2 deficiency significantly inhibited the expression of IFN-α. These results confirmed that high expression of IFN-α in Wip1^−/−^MSCs was related to endogenous BST2 expression.

IFN-α, encoded by the type 1 interferon gene, is known to be crucial for the pathogenesis of autoimmunity [34, 35], and contributed to the evolution of autoimmunity causing damages to β cells. Moreover, previous studies indicated MSCs could preferentially home to damage sites in certain tissues, which then enhanced wound healing and tissue regeneration, and inhibited the inflammatory reprocess [36, 37]. We revealed that IFN-α was highly expressed in Wip1^−^^/^^−^MSCs. Wip1^−/−^MSCs also could home to the pancreas in T1DM mice, leading to an increase of IFN-α, IL-17a, and TNF-α, and decrease of IL-4 and IL-10 in the pancreas, which exacerbated the inflammatory response and deterioration of clinical symptoms in T1DM mice. Therefore, our studies, together with previous investigations, demonstrated that there was high expression of IFN-α in Wip1^−/−^MSCs, which was mediated by BST2. However, in our study, the exact mechanisms of the Wip1-BST2-IFN-α axis in MSCs are still not well defined, which requires further investigation.

In summary, Wip1 deletion contributed to IFN-α expression in MSCs via increasing the expression of BST2, which thereby impaired the therapeutic effects of MSCs in T1DM mice. Wip1^−/−^ MSCs migrated to the pancreas in T1DM mice, which led to secretion of IFN-α and augmentation of inflammatory processes. Additionally, we indicated the importance of the Wip1-BST2-IFN-α axis in the therapeutic effects of MSCs in T1DM mice.

## Conclusions

This study identified the role of the Wip1 in regulating the immunomodulatory capabilities of MSCs. Furthermore, Wip1 was shown to directly impact the immunosuppression effect of MSCs in T1DM mice. The mechanism of Wip1 in MSCs immunomodulatory properties is pivotal for claiming the Wip1-BST2-IFN-α axis in the therapeutic effects of MSCs in T1DM mice.

## Materials and methods

### Mice

C57BL/6j mice (6–8 weeks, 20 ± 0.8 g) were purchased from Vital River Laboratory Animal Technology (Beijing, China). Wip1^−/−^mice were raised in a specific pathogen-free (SPF) room in the Advanced Laboratory Animal Center, the Academy of Military Medical Sciences (Beijing, China). All animals were handled according to the Guide for the Care and Use of Laboratory Animals. The protocol was approved by the Committee on the Ethics of Animal Experiments, the Academy of Military Medical Sciences.

### T1DM induction and experimental therapies

To induce the T1DM model as previously described [38, 39], male C57BL/6j mice were fed ad libitum for 48 h followed by food and water deprivation for 10–16 h before T1DM induction. Subsequently, streptozotocin (STZ, 50 mg/kg, Sigma-Aldrich, v900890) dissolved in sodium citrate buffer was administrated by intraperitoneal injection in the lower left abdomen once per day for 5 days. The ad libitum diet was resumed immediately after the first injection. T1DM was defined as a blood glucose of ≥16.7 mmol/L for three consecutive random samplings.

After T1DM induction, Wip1^+/+^MSCs (5 × 10^5^), Wip1^−/−^ MSCs (5 × 10^5^), and PBS (0.2 ml) were intravenously administered to T1DM mice on day 1 and 14, respectively (*n* = 10, three independent experiments). After treatment, the morphology, activity, fur, and food and water intake were monitored for each mouse. Random blood glucose and body weight were measured per week.

### Cell preparation

Murine primary MSCs were isolated from mouse compact bone and identified according to the previous description [40]. One-week-old mouse pups were collected and tailed for genotyping. The femur and tibia of Wip1^+/+^ and Wip1^−/−^ mice were isolated under aseptic conditions. The bone fragments were digested using type II collagenase at 37 °C for 45 min. Afterward, the mixture was transferred to α-MEM complete medium containing 10% fetal bovine serum. Adherent cells were maintained with medium replenishment every 3 days. The third passage cells were used for measuring differentiation characterization.

### Flow cytometry

Flow cytometry was used for the analysis of cell surface markers [41]. Cells were resuspended using PBS, and then were labeled with antibodies including PE anti-mouse MHCII (11-5321-81), FITC anti-mouse CD11b(RM2801), FITC anti-mouse Sca-1(11-5981-82), FITC anti-mouse CD34(11-0349-42), FITC anti-mouse CD45(11-0451-82), PE anti-mouse CD31(12-0311-82), PE anti-mouse CD29(12-0291-82), PE anti-mouse CD90(12-0909-42), or PE rat Ig2a isotype control (12-4301-81), or FITC rat Ig2a isotype control (PA5-33193) were purchased eBioscience. Subsequently, the cells were incubated in dark for 30 min, followed by washing with PBS. Cells were examined by flow cytometry with a FACS Calibur system [42].

For intracellular staining, splenic lymphocytes were collected from mice and stimulated for 4–6 h with Cell Stimulation Cocktail (eBioscience, 00-4970-03c), then the cells were stained with FITC-CD3 (eBioscience,11-0032-80) and PE-CD4 (eBioscience, MCD0404) for 30 min at 4 °C. Cells were then fixed and permeabilized using the Fixation/Permeabilization Diluent (eBioscience, 00-5223-56), according to the manufacturer’s instructions. For cytokine staining, the cells were stained with APC-IFN-γ (eBioscience, 17-7311-82) and measured with flow cytometry [43]. Data were analyzed with FlowJo.

### MSCs staining

Wip1^+/+^MSCs and Wip1^−/−^MSCs were harvested after centrifugation, followed by aspirating the supernatant. Then the cells were resuspended gently in CellTracker™ CM-Dil (1:1000, Invitrogen, C7000) staining solution, followed by incubating for 30 min at 37 °C. The stained cells resuspended with PBS were injected into T1DM mice.

### Histology analysis

Pancreatic sections were prepared from at least six mice in each group for hematoxylin-eosin (H&E) staining. Mouse insulin antibody (1:100; Cell Signaling Technology, 3014) was used for immunohistochemical and immunofluorescent staining of sections.

### Cell transfection

EGFP-Wip1 and EGFP plasmids (1 μg) were transiently transfected into 293 T cells (ATCC) using jetPRIME (polyplus,11415) for 48 h. Transfection efficiency was measured by flow cytometry. Wip1^−/−^MSCs (1 × 10^5^/well) were seeded into six-well plates. Then BST2 siRNA and negative control (50 nM, Sangon Biotech) were transfected into the cells using jetPRIME for 48 h.

### Gene knockdown using siRNA

BST2 expression was knocked down in Wip1^−/−^ MSCs using BST2-targeting siRNA. The siRNA target sequences for BST2 were: 5′-AGG CCG AGA CAC AGG CAA ATT-3′; 5′-AGG AGU CCC UGG AGA AGA ATT-3′; 5′-GAG AAU CUG AGG AUC CAA ATT-3′. The siRNA target sequences were transfected into Wip1^−/−^MSCs.

### Cytokine measurement

Levels of TNF-α, IFN-α, IL-4, and IL-10 in pancreas supernatant and IFN-γ, IL-4, and IL-17a in the serum of T1DM mice treated with Wip1^+/+^MSCs or Wip1^−/−^MSCs were determined by ELISA (Invitrogen, CA, USA). The level of IFN-α in Wip1^+/+^MSCs and Wip1^−/−^MSCs culture supernatant was measured by ELISA kits, according to the manufacturer’s instructions.

### Western blotting

Total protein was extracted from the cell pellet with RIPA lysis buffer (eBioscience, 89901). The protein concentration was determined by the BCA method using a commercial kit (Bio-Rad, M60-009RDPD). Protein lysis was separated by SDS-PAGE gel and then was transferred to a PVDF membrane. The PVDF membrane was blocked for 2 h in TBST buffer containing 5% skim milk powder and then tested with WiP1 Rabbit mAb (CST, 11901), BST2 Rabbit mAb (ABclonal, A12315), and β-Actin Rabbit mAb (CST,4970). Proteins were visualized using HRP-conjugated secondary antibodies. The bands were exposed by ECL software.

### Co-immunoprecipitation (Co-IP) assay

MSCs were rinsed twice with PBS, and lysed with RIPA lysis buffer (eBioscience, 89901) on 4 °C for 30 min, followed by centrifugation at 12,000×*g* for 15 min. Lysis supernatants were incubated with Wip1 Rabbit mAb or control IgG (Abcam, ab190475) overnight on a rotator, followed by the addition of 30 μl prewashed protein A/G agarose beads (CWBIO, CW0349S) for another 2 h. After extensive washing with a diluted lysis buffer, the lysate was used for western blot analysis.

### Quantitative real-time PCR (qPCR)

Total RNA was extracted from cultured cells using the TRIzol Reagent (Invitrogen, 15596018) and reverse-transcribed using SuperRT cDNA Synthesis Kit (CWBIO, CW0741). qPCR amplification was performed on a 7500 Real-Time system. The qPCR primers (1.0 OD per vial) purchased from Sangon (Sangon Biotech) were listed in Table S2. Relative quantification was performed using mouse-specific GAPDH primers.

### Statistical analysis

Data were presented as mean ± standard error of mean (SEM). The statistical significance of the differences between treatment groups was analyzed by Student’s *t*-test or one-way analysis of variance. Data analysis was performed using GraphPad Prism Version 6.0 software. *P* < 0.05 was considered to be statistically significant.

## Supplementary information

Figure S1

Table S1

Table S2

Supplementary Figure Legends

## Data Availability

All data generated or analyzed during this study are included in this published article and its supplementary information files.
