# Supplementary figures and images for: Wip1 regulates the immunomodulatory effects of murine mesenchymal stem cells in type 1 diabetes mellitus via targeting IFN-α/BST2
Source: Cell Death Discov. 2021 Oct 29;7:326. doi: 10.1038/s41420-021-00728-1 (PMC8556269; doi:10.1038/s41420-021-00728-1)

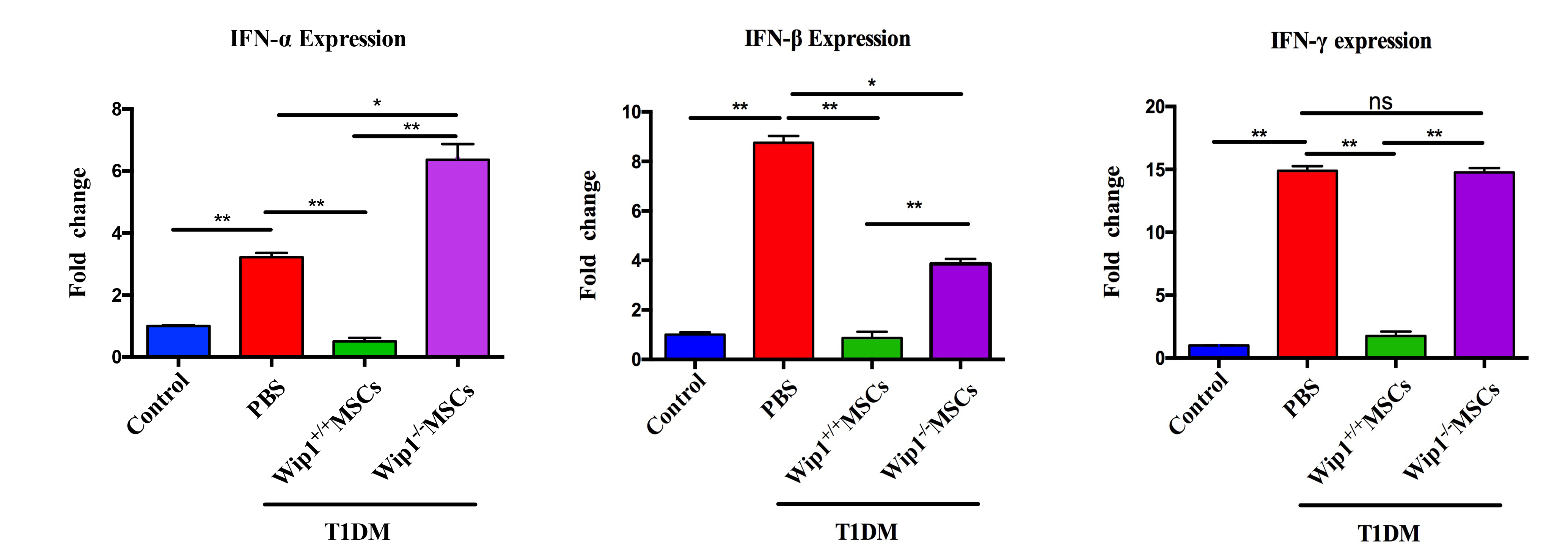

Supplement: Supplementary file 1 — Figure S1 [file 41420_2021_728_MOESM1_ESM.tif]
