# Supplementary material for: Wip1 regulates the immunomodulatory effects of murine mesenchymal stem cells in type 1 diabetes mellitus via targeting IFN-α/BST2
Source: Cell Death Discov. 2021 Oct 29;7:326. doi: 10.1038/s41420-021-00728-1 (PMC8556269; doi:10.1038/s41420-021-00728-1)
Supplement: Supplementary file 3 — Table S2 [file 41420_2021_728_MOESM3_ESM.docx]

Table 2. Primer sequences used in the Real-Time PCR

| Gene | Forward Primer (5’-3’) | Reverse Primer (5’-3’) |
| --- | --- | --- |
| *GAPDH* | ACTCTTCCACCTTCGATGC | CCGTATTCATTGTCATACCAGG |
| *GAS6* | GTGGCAAACTATCTCCGTG | ACCGCGATCTTCATTACAG |
| *Fabp3* | CATGTGCAGAAGTGGAACG | ATGAGTGAGAGTCAGGATGAG |
| *Prl2c3* | GACACATTTGAATTAGCCGG | AGAAGAGCTGCATAGTGTG |
| *IRAK4* | GATTTGGAGTGGTGTACAAGG | TACTGATTTCAACCATCGCTC |
| *TLR11* | GCTCAAAGAATCGATGCCA | CTCACCAGAGTCAATAGTATCA |
| *CCL19* | AGATTATCTGCCATGGCCC | TTGCCTTTGTTCTTGGCAG |
| *CCL6* | GGCCTCATACAAGAAATGGA | CTGAAGAAGTGTCTTGAAAGC |
| *KLRG-1* | GCTCTATCTATTCAACACTAGA | GTAAGACAGCTTTGAGCTTCC |
| *BST2* | AAGGAGCTTGAGAATGAAGTC | CACTGTGCTAGAAGTCTCC |
| *SH2D1B1* | CTGACCAAGCGAGAGTGTGA | TCCTCTTTGGCATAGGTTGT |
| *Cgas* | CATGTGAAGATTTCTGCTCCT | TCACAAGATAGAAAGCACCTG |
| *Src* | CTCCAGATTGTCAACAACAC | AAATACCACTCCTCAGCCT |
| *IFN-α* | TGTGACCTTCCTCAGACTC | TCATTTGTACCAGGAGTGTC |

Primers (1.0 OD per vial) were purchased from Sangon Biotech.
