# Supplementary material for: Wip1 regulates the immunomodulatory effects of murine mesenchymal stem cells in type 1 diabetes mellitus via targeting IFN-α/BST2
Source: Cell Death Discov. 2021 Oct 29;7:326. doi: 10.1038/s41420-021-00728-1 (PMC8556269; doi:10.1038/s41420-021-00728-1)
Supplement: Supplementary file 4 — Supplementary Figure Legends [file 41420_2021_728_MOESM4_ESM.docx]

Fig S1. Enhanced expression of *IFN-α, IFN-β, and IFN-γ* in T1DM mice spleen with infusion of Wip1^-/-^ MSCs.

The mRNA levels of *IFN-α, IFN-β,* and *IFN-γ* in splenic lymphocytes of T1DM mice were detected by qPCR. *P < 0.05, **P < 0.01.

Table S1. The microarray assay results of Wip1^+/+^MSCs and Wip1^-/-^MSCs.

Table S2. Primer sequences.
